# Dupilumab improves outcomes in patients with chronic rhinosinusitis with nasal polyps irrespective of gender: results from the SINUS‐52 trial

**DOI:** 10.1002/cti2.1511

**Published:** 2024-06-08

**Authors:** Wytske J Fokkens, Claus Bachert, Claire Hopkins, Osama Marglani, Amy Praestgaard, Scott Nash, Yamo Deniz, Paul J Rowe, Harry Sacks, Juby A Jacob‐Nara

**Affiliations:** ^1^ Department of Otorhinolaryngology Amsterdam University Medical Centres Amsterdam The Netherlands; ^2^ Department of Otorhinolaryngology – Head and Neck Surgery University Hospital of Münster Münster Germany; ^3^ International Airway Research Center First Affiliated Hospital, Sun Yat‐sen University Guangzhou China; ^4^ Department of Otorhinolaryngology King's College London London UK; ^5^ Department of Ophthalmology and Otolaryngology – Head and Neck Surgery Umm Al‐Qura University Makkah Saudi Arabia; ^6^ King Faisal Specialist Hospital and Research Center Jeddah Saudi Arabia; ^7^ Department of Biostatistics Sanofi Cambridge MA USA; ^8^ Medical Affairs Regeneron Pharmaceuticals Inc. Tarrytown NY USA; ^9^ Global Medical Affairs Sanofi Bridgewater NJ USA

**Keywords:** chronic rhinosinusitis, health‐related quality of life, patient‐reported outcomes, *post hoc* analysis

## Abstract

**Objectives:**

This *post hoc* analysis assessed disease characteristics and response to dupilumab treatment in male and female patients with severe chronic rhinosinusitis with nasal polyps (CRSwNP) (SINUS‐52 study; NCT02898454).

**Methods:**

Patients received dupilumab 300 mg or placebo every 2 weeks for 52 weeks on background intranasal corticosteroids. Efficacy was assessed through Week 52 using nasal polyp score (NPS), nasal congestion/obstruction score, loss of smell score and University of Pennsylvania Smell Identification Test score. Disease‐specific health‐related quality of life (HRQoL) was assessed using the 22‐item Sino‐Nasal Outcome Test (SNOT‐22).

**Results:**

The analysis included 192 male and 111 female patients. Female patients had higher mean SNOT‐22 total score (56.6 vs. 49.1, *P* < 0.01) and more coexisting asthma (78.4% vs. 46.4%, *P* < 0.0001) and non‐steroidal anti‐inflammatory drug‐exacerbated respiratory disease (NSAID‐ERD) (38.7% vs. 18.8%, *P* = 0.0001) than male patients, but other baseline characteristics were similar. Dupilumab significantly improved CRSwNP outcomes vs. placebo at Week 52, regardless of gender: least squares mean differences (95% confidence interval) for NPS were −2.33 (−2.80, −1.86) in male and −2.54 (−3.18, −1.90) in female patients (both *P* < 0.0001 vs. placebo), and for SNOT‐22 were −19.2 (−24.1, −14.2) in male and −24.4 (−31.5, −17.3) in female patients (both *P* < 0.0001 vs. placebo). There were no significant efficacy‐by‐gender interactions.

**Conclusion:**

Female patients had greater asthma, NSAID‐ERD and HRQoL burden at baseline than male patients. Dupilumab treatment significantly improved objective and subjective outcomes compared with placebo, irrespective of gender.

## Graphical abstract

This *post hoc* analysis of SINUS‐52 (NCT02898454) assessed disease characteristics and response to dupilumab treatment in male and female patients with severe chronic rhinosinusitis with nasal polyps. The analysis found female patients had greater asthma, non‐steroidal anti‐inflammatory drug‐exacerbated respiratory disease and health‐related quality‐of‐life burden at baseline than male patients. Dupilumab treatment significantly improved objective and subjective outcomes compared with placebo through 52 weeks, irrespective of gender.

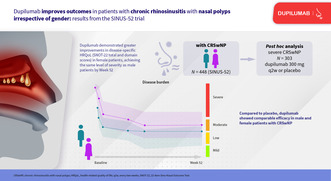

## Introduction

Chronic rhinosinusitis with nasal polyps (CRSwNP) is a chronic inflammatory disease of the nasal and paranasal sinuses, primarily driven by type 2 inflammation.^1^, ^2^, ^3^

The persistent and debilitating symptoms of CRSwNP, which include nasal congestion, rhinorrhoea and decreased/loss of sense of smell, profoundly impact patients' health‐related quality of life (HRQoL), with consequent negative effects on daily activities, mood, sleep and work productivity.^4^, ^5^, ^6^, ^7^ These symptoms frequently persist despite standard‐of‐care treatment with intranasal corticosteroids.

Sex‐ and gender‐related factors have been shown to affect the clinical presentation, outcomes and effects of therapies in a wide range of diseases^8^, ^9^; however, there is limited information on the impact of sex/gender in patients with CRSwNP. Studies have reported that the severity of certain symptoms of CRSwNP may be greater in female patients compared with male patients, and female patients experience worse general and disease‐specific HRQoL than their male counterparts.^10^, ^11^, ^12^, ^13^, ^14^ Differences in anatomic size, susceptibility to tobacco and hormonal factors have been speculated to increase the overall susceptibility to chronic rhinosinusitis in women compared with men.^11^, ^13^

Dupilumab blocks the shared receptor component for interleukin (IL)‐4 and IL‐13, which are central drivers of type 2 inflammation in CRSwNP and other inflammatory diseases.^1^, ^15^ The efficacy and safety of dupilumab in CRSwNP was demonstrated in the SINUS‐24 (NCT02912468) and SINUS‐52 (NCT02898454) randomised, placebo‐controlled trials.^16^ However, the efficacy of dupilumab by gender has not been studied in CRSwNP.

This *post hoc* analysis of the SINUS‐52 study assessed the baseline characteristics and response to dupilumab treatment in male and female patients with severe CRSwNP.

## Results

### Baseline characteristics

In the SINUS‐52 study, 448 patients were enrolled, with 150 receiving dupilumab q2w, 145 receiving at least one dose of dupilumab q2w for 24 weeks and q4w until Week 52 and 153 receiving placebo. This analysis included all 303 patients randomised to dupilumab 300 mg q2w or placebo, of whom 192 (63.4%) were male and 111 (36.6%) were female patients. Disease characteristics were generally similar in male and female patients (Table 1), although compared with male patients, a greater proportion of female patients had coexisting asthma (78.4% vs. 46.4%, *P* < 0.0001) and coexisting NSAID‐ERD (38.7% vs. 18.8%, *P* = 0.0001). Female patients had significantly higher baseline mean SNOT‐22 total score (56.6 vs. 49.1, *P* < 0.01) and nasal (3.3 vs. 3.0, *P* < 0.001) and ear/facial (1.8 vs. 1.3, *P* < 0.01) domain scores compared with male patients.

**Table 1:** Baseline demographics and disease characteristics by gender

	Male patients (*n* = 192)	Female patients (*n* = 111)	*P*‐value
Age, years	51.8 (12.4)	51.8 (12.2)	0.7950
Race, *n* (%)			
White	158 (82.3)	94 (84.7)	0.9407
Black	4 (2.1)	1 (0.9)	
Asian	23 (12.0)	12 (10.8)	
American Indian or Alaska Native	6 (3.1)	4 (3.6)	
Multiple	1 (0.5)	0	
Smoking history, *n* (%)			
Former	66 (34.4)	23 (20.7)	0.0135
Current	22 (11.5)	9 (8.1)	
Never	104 (54.2)	79 (71.2)	
Frequency of alcohol drinking in the past 12 months, *n* (%)			
Never	49 (25.5)	58 (52.3)	**< 0.0001**
Occasional	54 (28.1)	33 (29.7)	
At least monthly	20 (10.4)	7 (6.3)	
At least weekly	42 (21.9)	10 (9.0)	
At least daily	27 (14.1)	3 (2.7)	
Time since NP diagnosis, years	10.5 (9.30)	12.1 (10.8)	0.1781
Prior NP surgery, *n* (%)	111 (57.8)	65 (58.6)	0.8991
SCS use in previous 2 years, *n* (%)	158 (82.3)	85 (76.6)	0.2291
Coexisting asthma, *n* (%)	89 (46.4)	87 (78.4)	**< 0.0001**
Coexisting NSAID‐ERD, *n* (%)	36 (18.8)	43 (38.7)	**0.0001**
NPS (0–8)^a^	6.09 (1.16)	5.89 (1.30)	0.1527
Lund–Mackay score (0–24)^a^	17.88 (3.65)^b^	18.30 (3.78)	0.2987
NC score (0–3)^a^	2.43 (0.57)	2.42 (0.60)	0.9812
LoS score (0–3)^a^	2.74 (0.52)	2.81 (0.45)	0.5929
UPSIT score (0–40)^a^	13.72 (8.40)	13.45 (8.01)	0.8396
Anosmia, *n* (%)^a^ ^,^ ^c^	142 (76.3)	87 (79.8)	0.4897
SNOT‐22 total score (0–110)	49.10 (20.36)	56.57 (20.95)	**< 0.01**
Domain scores (0–5)^a^			
Nasal	2.96 (0.82)	3.30 (0.69)	**< 0.001**
Ear/facial	1.30 (1.15)	1.75 (1.30)	**< 0.01**
Sleep	2.29 (1.39)	2.52 (1.39)	0.1839
Function	2.06 (1.38)	2.36 (1.44)	0.1073
Emotion	1.63 (1.34)	2.00 (1.55)	0.0588

LoS, loss of smell; NC, nasal congestion/obstruction; NP, nasal polyp; NPS, nasal polyp score; NSAID‐ERD, non‐steroidal anti‐inflammatory drug‐exacerbated respiratory disease; SCS, systemic corticosteroid; SD, standard deviation; SNOT‐22, 22‐item Sino‐Nasal Outcome Test; UPSIT, University of Pennsylvania Smell Identification Test.

All data are mean (SD) unless otherwise stated. Significant *P*‐values are highlighted in bold.

^a^ Higher scores indicate greater disease severity, except for UPSIT, for which higher scores indicate lower disease severity.

^b^ *n* = 188.

^c^ Anosmia is defined as UPSIT < 19.

### Dupilumab efficacy: NPS and symptoms

**Figure 1:**
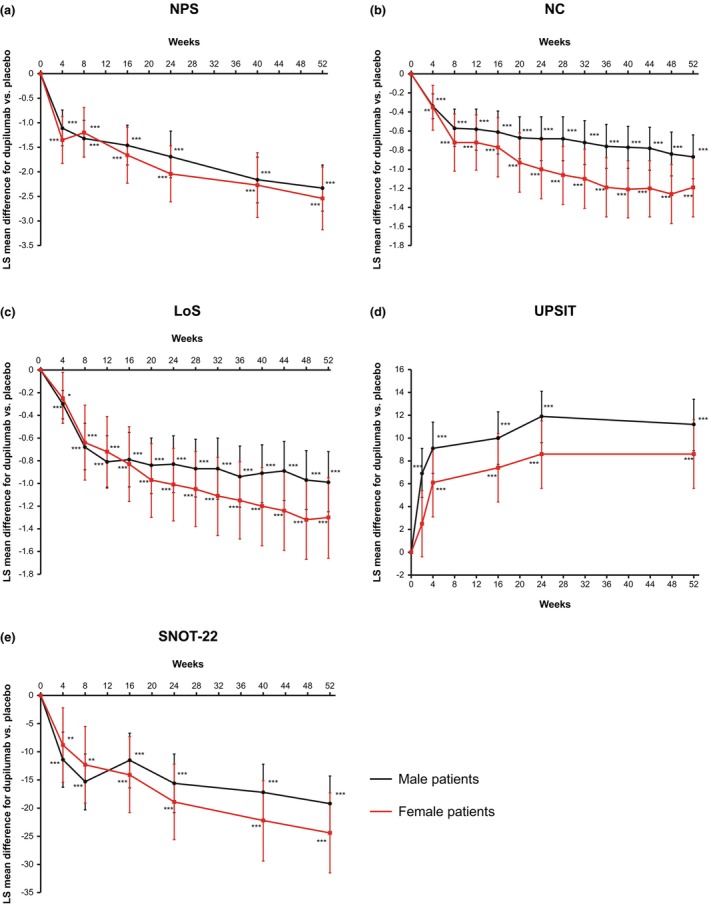
LS mean differences (95% CI) for dupilumab vs. placebo in change from baseline through Week 52 in **(a)** NPS, **(b)** NC, **(c)** LoS, **(d)** UPSIT and **(e)** SNOT‐22 by gender. Male patients, *n* = 95/97 (placebo/dupilumab); female patients, *n* = 58/53 (placebo/dupilumab). *P < 0.05, **P < 0.01, ***P < 0.0001 vs. placebo. CI, confidence interval; LoS, loss of smell; LS, least squares; NC, nasal congestion/obstruction; NPS, nasal polyp score; SNOT‐22, 22‐item Sino‐Nasal Outcome Test; UPSIT, University of Pennsylvania Smell Identification Test.

In both male and female patients, dupilumab significantly improved NPS and symptoms vs. placebo at Week 52; there was no significant gender‐by‐treatment interaction for any of the outcomes (Table 2). For efficacy outcomes, least squares (LS) mean differences (95% confidence interval [CI]) for dupilumab vs. placebo in the change from baseline at Week 52 were as follows: NPS, −2.33 (−2.80, −1.86) in male and −2.54 (−3.18, −1.90) in female patients; NC score, −0.87 (−1.10, −0.64) in male and −1.19 (−1.50, −0.88) in female patients; loss of smell (LoS) score, −0.99 (−1.26, −0.73) in male and −1.30 (−1.65, −0.94) in female patients; and UPSIT score, 11.2 (9.0, 13.5) in male and 8.6 (5.6, 11.6) in female patients (all *P* < 0.0001 vs. placebo). Significant improvements with dupilumab vs. placebo were evident from Week 4 through Week 52 for all efficacy outcomes in both male and female patients. However, the differences in efficacy outcomes between male and female patients were clinically insignificant (Figure 1).

**Table 2:** LS mean difference (95% CI) for dupilumab vs. placebo in change from baseline at Week 52 in NPS, NC, LoS, UPSIT and SNOT‐22 by gender

	Male patients (*n* = 95/97 [placebo/dupilumab])	Female patients (*n* = 58/53 [placebo/dupilumab])	Gender/efficacy interaction, *P*‐value
NPS (0–8)	−2.33 (−2.80, −1.86)***	−2.54 (−3.18, −1.90)***	0.5536
NC score (0–3)	−0.87 (−1.10, −0.64)***	−1.19 (−1.50, −0.88)***	0.5754
LoS score (0–3)	−0.99 (−1.26, −0.73)***	−1.30 (−1.65, −0.94)***	0.7426
UPSIT score (0–40)	11.24 (8.97, 13.51)***	8.62 (5.64, 11.61)***	0.3232
SNOT‐22 total score (0–110)	−19.15 (−24.12, −14.17)***	−24.39 (−31.45, −17.34)***	0.7143

CI, confidence interval; LoS, loss of smell; LS, least squares; NC, nasal congestion/obstruction; NPS, nasal polyp score; SNOT‐22, 22‐item Sino‐Nasal Outcome Test; UPSIT, University of Pennsylvania Smell Identification Test.

*** *P* < 0.0001 vs. placebo.

### Dupilumab efficacy: HRQoL

**Figure 2:**
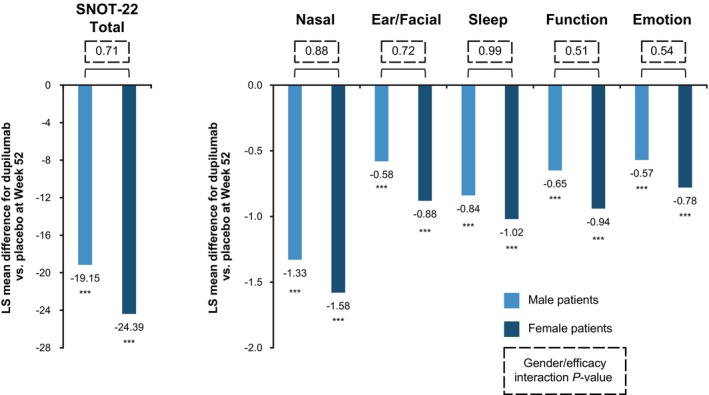
LS mean differences for dupilumab vs. placebo in change from baseline at Week 52 in SNOT‐22 total and domain scores by gender. ***P < 0.0001 vs. placebo. LS, least squares; SNOT‐22, 22‐item Sino‐Nasal Outcome Test.

Dupilumab significantly improved CRSwNP‐specific HRQoL, as measured by SNOT‐22, at Week 52 vs. placebo in both male (LS mean difference [95% CI] −19.2 [−24.1, −14.2]) and female patients (−24.4 [−31.5, −17.3]) (both *P* < 0.0001 vs. placebo); there was no significant gender‐by‐treatment interaction (Table 2). Significant improvement in SNOT‐22 total score vs. placebo was seen from the first assessment (Week 4) in both male and female patients (Figure 1). All five SNOT‐22 domain scores significantly improved with dupilumab vs. placebo at Week 52 in both male and female patients (LS mean differences: nasal, −1.33 male and −1.58 female; ear/facial, −0.58 and −0.88; sleep, −0.84 and −1.02; function, −0.65 and −0.94; and emotion, −0.57 and −0.78; all *P* < 0.0001 vs. placebo) (Figure 2). There was no clinically significant gender‐by‐treatment interaction for any of the domain scores.

## Discussion

Some differences in baseline disease characteristics were observed between male and female patients with severe CRSwNP who enrolled in the SINUS‐52 study. Compared with male patients, female patients in the current analysis had a higher prevalence of coexisting asthma (78% vs. 46%) and coexisting NSAID‐ERD (39% vs. 19%). These findings are consistent with a previous retrospective, single‐centre study that reported higher frequencies of comorbid asthma (66% vs. 46%) and NSAID‐ERD (13% vs. 5%) in female vs. male patients with CRSwNP.^13^

The baseline difference in HRQoL between male and female patients in the current analysis was statistically significant in the nasal and ear/facial domains of the SNOT‐22 score, suggesting that these aspects of HRQoL may specifically affect female patients more than male patients. Similar data were reported previously in a study of patients with chronic rhinosinusitis undergoing endoscopic sinus surgery, which found that female patients reported more problems with postnasal drainage and facial pain than male patients.^12^

Previous studies draw conflicting conclusions about the gender differences in CRSwNP. A retrospective review of patients with chronic rhinosinusitis (with and without nasal polyps) electing for endoscopic sinus surgery^12^ and a surgical cohort study^14^ reported a greater HRQoL impact in female vs. male patients with CRSwNP,^12^, ^14^ while a literature review^11^ and a recent retrospective, single‐centre cohort study^17^ observed no significant gender differences. Differences in HRQoL measures, patient cohorts and study settings may explain these variable findings. For example, patients with CRSwNP and comorbid asthma and/or NSAID‐ERD are known to have a greater disease burden and worse HRQoL than those without these comorbidities.^18^ There is also discordance in the literature regarding disease presentation in male and female patients with CRSwNP: one study showed no gender difference for NPS,^17^ whereas others report more severe nasal congestion^10^ and higher polyp scores^14^ in male vs. female patients with CRSwNP. In our analysis, disease characteristics between male and female patients were generally the same, with the exception of lower HRQoL and the higher prevalence of coexisting asthma and NSAID‐ERD in female patients.

Despite observed differences at baseline, dupilumab significantly improved SNOT‐22 total and domain scores over 52 weeks irrespective of gender, with female patients achieving similar levels of mild/moderate disease severity as male patients by Week 52. The trend for greater improvement in SNOT‐22 total and domain scores in female patients may reflect their higher baseline severity.

Loss of smell is one of the most troublesome and difficult‐to‐treat symptoms for patients with severe CRSwNP.^19^ In the general population, women are known to outperform men in olfactory ability,^20^ but whether this difference translates into differences in olfactory outcomes in CRSwNP is not known. The current study found no significant differences between male and female patients with respect to smell improvement with dupilumab assessed using UPSIT or as self‐reported by patients.

Overall, the results of this *post hoc* analysis show that, despite the higher HRQoL burden in female patients with CRSwNP, overall dupilumab efficacy was similar in male and female patients. The results add to the body of evidence demonstrating that dupilumab efficacy in CRSwNP is unaffected by a range of factors including patient phenotype,^21^, ^22^, ^23^ disease endotype^3^ and treatment history.^24^, ^25^

Disease characteristics were generally similar at baseline between male and female patients with CRSwNP, except that female patients had a greater asthma, NSAID‐ERD and HRQoL burden than male patients. Dupilumab treatment significantly improved objective and subjective outcomes compared with placebo, irrespective of gender.

## Methods

### Study design and patients

Full details for the study design and patient eligibility of SINUS‐52 have been published elsewhere.^16^ In brief, patients were randomised 1:1:1 to dupilumab 300 mg subcutaneous (SC) every 2 weeks (q2w) for 52 weeks, dupilumab 300 mg SC q2w to Week 24 and then every 4 weeks (q4w) to Week 52 or matching placebo for 52 weeks; all patients received daily background therapy with a stable dose of intranasal mometasone furoate nasal spray. The studies were conducted in accordance with Good Clinical Practice and with the principles ordained in the Declaration of Helsinki, the protocols were approved by appropriate ethical review boards and all patients provided written informed consent.

### Outcome measures

Patient demographics and disease characteristics were assessed at baseline. Efficacy was assessed through 52 weeks of treatment using nasal polyp score (NPS; range 0–8), nasal congestion/obstruction (NC) score (range 0–3), loss of smell (LoS) score (range 0–3) and the University of Pennsylvania Smell Identification Test (UPSIT) score (range 0–40). Disease‐specific HRQoL was assessed using the 22‐item Sino‐Nasal Outcome Test (SNOT‐22) total score (range 0–110) and domain scores (nasal, ear/facial, sleep, function and emotion; range 0–5 for each domain).^26^

### Statistical analyses

All analyses were conducted in patients treated with placebo or dupilumab 300 mg q2w from the intention‐to‐treat population. Baseline parameter *P*‐values were computed using a Chi‐square test for qualitative parameters. Baseline parameter *P*‐values were computed using Wilcoxon rank‐sum test for age, race, smoking and alcohol history quantitative parameters, and Kruskal–Wallis test for quantitative parameters. Differences in change from baseline between dupilumab and placebo, and interaction *P*‐values for male vs. female patients, were determined using analysis of covariance (ANCOVA) models. The interaction *P*‐value was computed by fitting an ANCOVA model with the corresponding baseline value, treatment group, asthma/non‐steroidal anti‐inflammatory drug‐exacerbated respiratory disease (NSAID‐ERD) status, prior nasal polyp surgery and regions as covariates, plus the subgroup variable and the subgroup‐by‐treatment interaction. The placebo group and male subgroup were considered as references, respectively, for the treatment and NPS groups. All reported *P*‐values are nominal.

## Conflict of interest

WJF receives research grants from BioInspire Technologies, GlaxoSmithKline, Meda Pharmaceuticals and Sanofi. CB is an advisory board member and receives speakers' fees from ALK, AstraZeneca, GlaxoSmithKline, Mylan, Novartis, Sanofi and Stallergenes Greer. AP, PJR and JAJ‐N are employees and may hold stock and/or stock options in Sanofi. CH is an advisory board member of AstraZeneca, Dianosic, GlaxoSmithKline and Sanofi. OM reports no conflicts of interest. SN, YD and HS are employees and may hold stock and/or stock options in Regeneron Pharmaceuticals Inc.

## Author contributions

**Wytske J Fokkens:** Formal analysis; investigation; writing – original draft; writing – review and editing. **Claus Bachert:** Formal analysis; investigation; writing – original draft; writing – review and editing. **Claire Hopkins:** Formal analysis; investigation; writing – original draft; writing – review and editing. **Osama Marglani:** Formal analysis; investigation; writing – original draft; writing – review and editing. **Amy Praestgaard:** Conceptualization; formal analysis; methodology; validation; writing – original draft; writing – review and editing. **Scott Nash:** Conceptualization; formal analysis; methodology; writing – original draft; writing – review and editing. **Yamo Deniz:** Conceptualization; formal analysis; methodology; writing – original draft; writing – review and editing. **Paul J Rowe:** Conceptualization; formal analysis; methodology; writing – original draft; writing – review and editing. **Harry Sacks:** Conceptualization; formal analysis; methodology; writing – original draft; writing – review and editing. **Juby A Jacob‐Nara:** Conceptualization; formal analysis; methodology; writing – original draft; writing – review and editing.

## Data Availability

Qualified researchers may request access to patient‐level data and related study documents including clinical study reports, study protocol with any amendments, blank case report form, statistical analysis plan and dataset specifications. Patient‐level data will be anonymised, and study documents will be redacted to protect the privacy of trial participants. Further details on Sanofi's data sharing criteria, eligible studies and process for requesting access can be found at: https://www.vivli.org/.
